# Robotic Ankle Assessment Post-Stroke: Reliability, Comparison to Therapists, and Benchmark Dataset Development

**DOI:** 10.3390/s25206405

**Published:** 2025-10-17

**Authors:** Christopher A. Johnson, Andria J. Farrens, Piyashi Biswas, Luis Garcia-Fernandez, Jill See, Lucy Dodakian, Vicky Chan, Po T. Wang, Steven C. Cramer, Zoran Nenadic, An H. Do, David J. Reinkensmeyer

**Affiliations:** 1 Rancho Los Amigos National Rehabilitation Center, Rancho Research Institute, Downey, CA 90242, USA; 2 Department of Orthopaedics and Rehabilitation, University of New Mexico, Albuquerque, NM 87131, USA; 3 Department of Biomedical Engineering, University of California Irvine, Irvine, CA 92697, USAdreinken@uci.edu (D.J.R.); 4 Department of Mechanical and Aerospace Engineering, University of California Irvine, Irvine, CA 92697, USA; 5 Irvine Medical Center, Department of Rehabilitation Services, University of California, Orange, CA 92868, USA; 6 Department of Neurology, University of California Los Angeles, Los Angeles, CA 90095, USA; 7 Department of Electrical Engineering and Computer Science, University of California Irvine, Irvine, CA 92697, USA; 8 Department of Neurology, School of Medicine, University of California, Irvine, Irvine, CA 92697, USA; 9 Department of Anatomy and Neurobiology, University of California Irvine, Irvine, CA 92697, USA

**Keywords:** test–retest reliability, ankle motor function, robotic assessment

## Abstract

In rehabilitation research, coarse clinical outcome measures may limit the sensitivity of trials by failing to detect meaningful intervention-induced changes. Sensorized robotic platforms can potentially improve the evaluation of motor function, yet their reliability compared to skilled therapists remains unclear. This study utilized a robotic device to measure two fundamental impairments that are critical to ankle function: range of motion (ROM, active and passive) and dorsiflexion maximum voluntary contraction (MVC). In 34 chronic hemiparetic post-stroke individuals, we assessed test–retest reliability over two days for the robot and experienced therapists, who used a goniometer and manual muscle testing (MMT). We also evaluated robotic test–retest reliability in 36 young and 26 older unimpaired adults. Reliability for robotic and therapist-based AROM and MVC measures was high (ICC > 0.86), consistent across all groups. Robotic AROM and PROM measurements correlated strongly with therapist assessments (r > 0.60, *p* < 0.001) but were 120% and 37% larger than therapist assessments (*p* < 0.001), respectively. For the MVC measurement, the therapist assigned 85% of participants a score of 1 on the MMT, but their MVC torque was distributed from 0 to ~20 Nm. Measurement differences between methods likely arose from the robot’s constrained setup, allowing for compensatory muscle activation. The increased granularity provided by robotic MVC measurements could enable more precise tracking of motor recovery and facilitate tailored rehabilitation strategies. These results support the clinical utility of robotic platforms for ankle assessment, offering detailed, objective measurements that can augment traditional evaluations, and provide a benchmark dataset for other researchers.

## 1. Introduction

Impaired gait following stroke is strongly associated with reduced independence, difficulty leaving the home, and limited community and social participation [1,2]. These limitations are often driven by impairments in ankle joint motion and strength, which are common after stroke and negatively impact gait and balance function [3,4,5]. Assessing ankle impairment is critical in diagnosing gait deficits, guiding treatment plans, and monitoring treatment progression [6,7].

Two key assessments often completed by physical therapists to assess ankle motor impairment are range of motion (ROM, both passive (PROM) and active (AROM)) and maximum voluntary contraction (MVC). The methods for measuring ankle ROM can be broadly classified into three categories: goniometry, weight-bearing, and instrumented techniques [8], but the most used technique in the clinic is the goniometer. Goniometers are inexpensive and convenient but require technical proficiency due to the necessity of aligning the axis with the joint fulcrum and positioning the two arms with established reference points, a process that is even more complicated during PROM measurements when the therapist must hold the goniometer while manually moving the joint [6,9]. For assessing MVC, there are many methods and tools, including handheld dynamometers, but the most used technique in the clinic is the Manual Muscle Test (MMT), as it is quick and easy to perform [10]. However, its reliability is low because its grading depends on subjective assessment of force, which can be influenced by the examiner’s muscle strength and temporal variations in force production [11].

With these challenges of assessing the ankle, alternative methods have been proposed, such as electromechanical sensing technologies [12]. Within the last couple of decades, there has been an increase in the use of robotic devices for neurorehabilitation training in clinical centers, including both wearable exoskeletons and platform-based devices [13,14,15]. Besides using them for movement training, translational researchers in neurorehabilitation have proposed using the sensors embedded in these robotic devices to overcome some of the limitations in traditional clinical assessments [16]. Sensorized robotic devices can potentially provide accurate (e.g., able to control/measure exact body position/force applied) and objective (not relying as strongly on observer judgement) measurements [13,16]. Yet there is currently limited information on the reliability and validity of such systems [13,17].

To address this gap, we used a bilateral, platform-based robotic device to evaluate two fundamental aspects of ankle function, dorsiflexion/plantarflexion ROM and MVC, in persons post-stroke. For comparison, skilled rehabilitation therapists acquired the same measures using the aforementioned most common clinical approaches to these assessments. We hypothesized that the robotic device would facilitate the acquisition of both reliable and valid measurements of ankle ROM and dorsiflexion MVC, comparable to those from the therapists.

## 2. Materials and Methods

### 2.1. Participants

Persons in the chronic phase post-stroke were enrolled in a clinical trial designed to evaluate the efficacy of a brain–computer interface combined with a functional electrical stimulation system for treating foot drop (clinicaltrials.gov NCT04279067). Clinical and functional status of stroke participants was characterized using standardized assessments, as shown in Table 1. For this analysis, only ROM and MVC measurements taken at baseline and prior to the start of therapy were included. These measurements were spaced one week apart.

For an extensive list of inclusion and exclusion criteria, see [18], but here, we list several key inclusion criteria: (1) age, 18–80 years inclusively at time of consent; (2) radiologically confirmed bilateral or unilateral stroke, ischemic or intracerebral hemorrhage (ICH) in etiology, with day of onset at least 26 weeks prior to day of randomization; (3) gait velocity < 0.8 m/s at screening and baseline visits; (4) foot drop in affected limb as defined by dorsiflexion active range of motion (AROM) via goniometry in seated position foot dangling is less than passive range of motion and less than 15 degrees; (5) can walk > 10 m (with or without AFO, and cane or walker permitted) at a supervised level. Exclusion criteria included the following: (1) a major, active, coexisting medical, neurological (apart from stroke), or psychiatric disease (apart from stroke); (2) implanted electronic device (e.g., pacemaker) or skull metallic implants; (3) pregnancy; (4) significant pain (visual analog scale > 4), chest pain, or shortness of breath with walking; (5) non-English speaking, such that participant does not speak sufficient English to comply with study procedures; (6) received chemical denervation (e.g., botulinum toxin) to legs in the preceding 6 months; (7) significant cognitive impairment, defined as Montreal Cognitive Assessment score (MoCA) < 22—as MoCA scores for those with aphasia may be difficult to interpret, this exclusion criterion may be waived at the discretion of the clinical team. The local ethics committee approved this study, and written informed consent was obtained from each participant prior to participating, following the procedures established by the University of California, Irvine Institutional Review Board.

For comparison, young uninjured participants, 18–35 years old, and older uninjured adults with ages selected to match the average age of our stroke participants were recruited for two assessment sessions. For young and older unimpaired participants, the exclusion criteria were as follows: history of neurological injury, musculoskeletal damage to the ankles, current injuries that affected participants’ ability to move or feel either of their ankles, or use of medication that would change how the brain perceived pain/movement. Leg dominance was determined using a self-report question from the method described by van Melick et al., in which participants indicate which leg they would use to kick a ball [19].

### 2.2. Robotic Device

**Figure 1:**
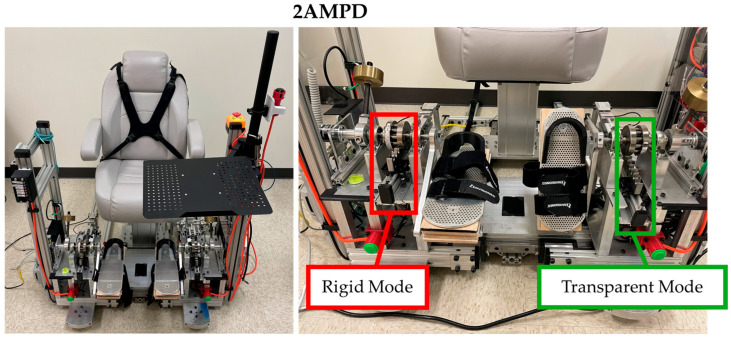
Device used to measure ankle active range of motion, passive range of motion, and dorsiflexion maximum strength. In rigid mode, the foot pedal is coupled to a non-backdriveable, linear actuator so that isometric force can be measured or the ankle can be passively driven. In transparent mode, the pedal is decoupled from the drive, allowing free ankle dorsiflexion/plantarflexion movement.

Two versions of the Ankle Measuring Proprioceptive Device (AMPD and 2AMPD) were used for this study. 2AMPD is an improved version of AMPD that was designed to improve the ease of participant transfers to the device by lowering the seat height. Here, we briefly describe the common features of both AMPD and 2AMPD [20]. Each device has two modes: rigid and transparent, terms that describe how the foot pedal feels when the participant pushes against it. The modes are selected by operating a mechanical clutch (Figure 1). In its rigid mode, the device can individually assist and move both ankles, via linear actuators (lead screw actuators) and a rack and roller pinion system, through participants’ natural dorsiflexion and plantarflexion passive range of motion. In transparent mode, the rack and roller pinion is mechanically disconnected, enabling voluntary ankle movement with minimal impedance. This is achieved through low-friction ball bearings around which the footplates rotate and a counterbalancing system that neutralizes gravity. The devices are equipped to measure ankle position (dorsiflexion and plantarflexion), via quadrature angular encoders (0.88° resolution, Sparkfun, Boulder, CO, USA), and ankle force converted to ankle torque, via s-type load cells (Interface SMA, 200 lbf/900 N, Scottsdale, AZ, USA). Sample frequency was set to 200 Hz, and data was stored on a laptop.

Prior to the start of the study, each footplate underwent static calibration. Known weights were hung from the footplate, load-cell output (mV) was recorded at each load, and linear regressions were derived to obtain the equation to convert mV into kg. Torque was then computed as τ=r F cos θ, where r is the moment arm from the center of the shaft to the front bolt of the foot pedal, and θ is the encoder-measured ankle angle. For all robotic data, signals were filtered with a low-pass (10 Hz cutoff) 4th-order Butterworth filter. To eliminate phase distortion, filtering was applied in a zero-phase forward–backward manner.

### 2.3. Robotic Assessments

For all participants, ROM and MVC were measured in two sessions that were three to ten days apart, using AMPD devices. Participants were assigned to a single device version (AMPD or 2AMPD) and returned to the same unit for the second session; no crossover between devices occurred. Both sessions were scheduled at the same time of day (morning or afternoon), within two hours of the first session’s start time. For post-stroke participants, no treatment occurred between the first and second assessments.

For the post-stroke participants, two skilled physical therapists (PTs), each with 20+ years of experience in assessing motor function after stroke, used AMPD to assess ROM and MVC. Robotic assessments were always performed after therapist assessments during each session. Within each robotic session, AROM was assessed first, followed by dorsiflexion MVC, and then passive ROM. For young and older unimpaired adults, a trained operator assessed AROM and MVC in this exact order.

Using AMPD, all participants sat in an upright position with their hips and knees bent at 90 degrees such that the shanks were perpendicular to the ground and the feet were hip-width apart. The feet were strapped to the AMPD footplates after wood shims were added to the footplates to align the lateral malleolus with the rotational shaft of AMPD. Once participants were in the correct seated position, the chair position was recorded, using built-in distance sensors (±1 mm resolution), and saved so it could be used for the second session of measurements. Standardized instructions and a demonstration were provided before each ankle test. Prior to each session, the footplate was leveled to subtalar joint neutral (STN) using a bubble level, and the encoder was zeroed.

To measure ROM, AMPD was placed in its transparent mode. For the AROM measurements, participants were instructed to move their ankle to the maximum dorsiflexion angle without lifting the heel off the foot pedal and then transition to their maximum plantarflexion angle without internally rotating the hip. A single trial consisted of each maximum angle held for ~3 s. All participants performed three trials on each ankle, and 15 s of rest was given between each trial. For stroke participants, three trials were performed on the unimpaired ankle first to aid in understanding the task, and then, the unimpaired ankle was measured. To measure PROM, only the impaired ankle was measured by the PT, who manually rotated the impaired ankle foot pedal into dorsiflexion until the first end-feel resistance was encountered without inducing discomfort. First end-feel corresponds to the point of appreciable passive resistive torque in the ankle, aligning with clinical guidance on end-feel at the end-range and with the ankle literature [21,22]. That ankle angle was saved using a button, and only one trial was completed.

To measure dorsiflexion MVC, AMPD was placed in its rigid mode with the ankles locked at an angle of 90° in the parasagittal plane. Participants were asked to gradually dorsiflex one ankle until they reached maximum effort and hold for three seconds. Like AROM measurements, participants performed three trials on each ankle. For participants who were post-stroke, three trials were performed on the unimpaired ankle first to aid in understanding the task, and then, the impaired ankle was measured. Because AROM and MVC measures are effort-dependent and can fluctuate across attempts, we collected three trials per measure to obtain a stable estimate in line with common practice [23,24,25]. PROM does not depend on participant effort, so a single therapist-driven trial to firm resistance was recorded. The peak values for AROM and MVC measurements were used as their maximum ankle angle or torque, respectively.

### 2.4. Clinical Assessments (Only Stroke)

For the post-stroke participants, the same two PTs also manually assessed the dorsiflexion ROM (both PROM and AROM) and MVC. AROM and PROM were assessed before MVC. The test order was kept consistent across all participants and both sessions. While this fixed ordering facilitated standardization, it also introduced a potential ordering effect, which was not counterbalanced or randomized. The post-stroke participants were seated on a gurney in an upright position with the hip and knee bent at 90 degrees such that the shank was perpendicular to the ground and the feet suspended in the air.

To measure the dorsiflexion AROM using the goniometer, the PT positioned the goniometer so that the rotation axis rested over the center of the lateral malleolus. They aligned the stationary goniometer arm parallel to the longitudinal axis of the fibula and the mobile arm parallel to the longitudinal axis of the fifth metatarsal bone. The therapist measured the impaired ankle dorsiflexion AROM three times to the nearest degree. A similar procedure was used to measure PROM, but in this case, the therapist manually dorsiflexed the ankle with their knee to the felt end of the ROM [26], and three trials were performed.

The manual muscle test (MMT) was used to measure dorsiflexion MVC. Each stroke participant was asked to dorsiflex as strongly as possible, and the PT rated the force generated using the Medical Research Council Scale (0–5) [27]. Only one trial was performed.

### 2.5. Statistical Analysis

Statistical analyses were conducted using MATLAB R2023 and the JMP Pro 16 software. For dorsiflexion MVC, if participants did not overcome the weight of their foot to create a positive flexion torque, they received a zero-dorsiflexion torque for that specific trial. We did not perform a formal power analysis. However, our sample size (young, n = 36; age-matched controls, n = 26; stroke, n = 34) is consistent with ICC design recommendations and exceeds sample sizes used in comparable ankle reliability studies [9,23,24], supporting the adequacy of our estimates. The intraclass correlation coefficient (ICC) with 95% confidence intervals (CIs) was calculated using two-way mixed effects with a single rater [28] to assess the test–retest reliability (session 1 compared to session 2 measurements) for each. ICC values less than 0.5 indicate poor reliability, values between 0.5 and 0.75 indicate moderate reliability, values between 0.75 and 0.9 indicate good reliability, and values greater than 0.90 indicate excellent reliability [28]. Standard error of measurement (SEM) was calculated using the standard deviation (SD), where SEM = SD ∗ $\sqrt{1-ICC}$, and minimal detectable change (MDC) at 95% confidence level was computed using the formula MDC = SEM ∗ 1.96 ∗ √2 [29].

Each output parameter (AROM and MVC) for each group, using the average of 3 trials, was independently tested for normality using the Shapiro–Wilk test. All AROM and MVC measurements were normally distributed (*p* > 0.05), justifying the use of parametric tests. To assess validity, Pearson’s correlation coefficient was calculated between robotic and physical therapist measurements. Paired *t*-tests were used to compare validation measurements.

For chronic stroke participants, a repeated-measures ANOVA was conducted to examine the main effects of timepoint (first vs. second session) and ankle impairment (impaired vs. unimpaired side), stratified by sex. For comparisons using sex, we compared only the same sex across groups. For unimpaired participants, repeated-measures ANOVA was used to assess the effects of timepoint (first vs. second session), leg dominance (dominant vs. nondominant), age group (young vs. older), and sex (female vs. male). If significant main or interaction effects were identified, post hoc comparisons were performed using Tukey’s Honest Significant Difference (HSD) test. Effect sizes were calculated using Cohen’s *d*, with values of 0.2, 0.5, and 0.8 interpreted as small, medium, and large effects, respectively [30]. The level of statistical significance was set at *p* < 0.05.

## 3. Results

In total, 34 persons in the chronic phase of stroke (19 male, 15 female; age = 60 ± 12 yrs) participated in ankle ROM and MVC assessments during two sessions that were, on average, 7 ± 2 days apart. In addition, 36 young, unimpaired participants (23 male, 13 female; mean ± SD, age = 25 ± 4 yrs) and 26 older, unimpaired participants (10 male, 16 female; age = 65 ± 10 yrs) completed the same protocol for robotic assessments. The older group was age-matched to the stroke group (*p* = 0.16, *t*-test), and the percentage of males was similar in each group (56% vs. 38%). The ankle ROM and MVC of participants who were post-stroke were assessed using a sensorized robotic platform (AMPD) and by a physical therapist (PT) using a goniometer and manual muscle testing (MMT). Clinical characteristics of the participants who were post-stroke are shown in Table 1. Demographics of the unimpaired participants are shown in Table 2.

### 3.1. Overview of Measurements and Effect of Stroke

**Figure 2:**
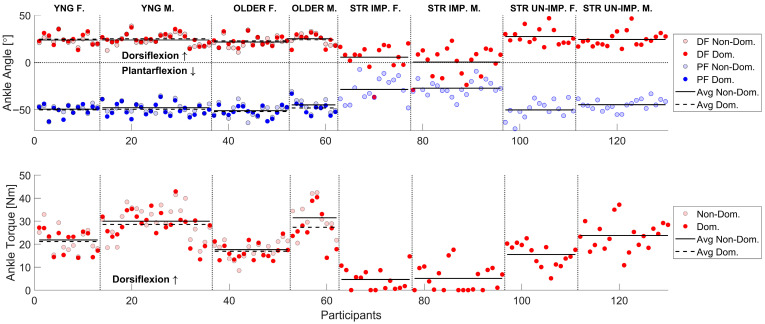
Averages per participant for AROM and dorsiflexion MVC obtained using the sensorized robotic platform, AMPD, for all groups at Session 1 but stratified by sex and leg dominance. **Top**: AROM dorsiflexion and plantarflexion angles for the dominant and non-dominant ankle. **Bottom**: Dorsiflexion MVC for the dominant and non-dominant ankle. The dotted and solid black lines represent the averages for the dominant and non-dominant ankle. DF: dorsiflexion, PF: plantarflexion, Non-Dom: non-dominant, Dom: dominant, YNG F: young female, YNG M: young male, OLDER F: older female, OLDER M: older male, STR IMP. F: stroke-impaired side female, STR IMP. M: stroke-impaired side male, STR UN-IMP. F: stroke-unimpaired side female, STR UN-IMP. M: stroke-unimpaired side male.

Figure 2 provides an overview of the AROM and dorsiflexion MVC measurements collected during the first session using AMPD for all participants, while Table 3 summarizes the averages. Viewing the data in Figure 2 provides a sense of the inter-subject variability in these measurements, as well as the decreased AROM and dorsiflexion MVC of the hemiparetic ankle. A supplemental data file provides the corresponding numerical measurements, along with basic demographic and clinical descriptors of the participants, for use as a benchmark in future studies.

Within the stroke participants, the impaired ankle had significantly less AROM and dorsiflexion strength compared to the unimpaired ankle, as measured with the robot (*p* < 0.0001, d > 2.0). Stroke participants’ unimpaired AROM was comparable to that of unimpaired age-matched controls (*p* > 0.4). However, they generated significantly less dorsiflexion torque on their “unimpaired” side compared to controls (*p* < 0.001, d = 0.3).

### 3.2. Test–Retest Reliability of Robot- and Therapist-Based Measurement of Ankle ROM and MVC

As described in the Methods, therapist test–retest data were collected for the impaired ankles of stroke participants for dorsiflexion AROM and MVC (and not plantarflexion or passive ROM). Both the robot and therapist measurements of ankle dorsiflexion AROM and MVC showed excellent test–retest reliability: ICCs were always 0.91 or above for AROM, and 0.86 or above for MVC (Table 4).

We additionally assessed reliability via the minimal detectable change (MDC) estimated from the two AROM or MVC measurements (Table 3). The MDC resulting from the robot measurements was typically lower than for the therapist, a fact attributable to the greater variability in the two measurements obtained with AMPD for both AROM and MVC (see SEM, Table 4).

### 3.3. Validity of Robot-Based Compared to Therapist-Based Measurement

**Figure 3:**
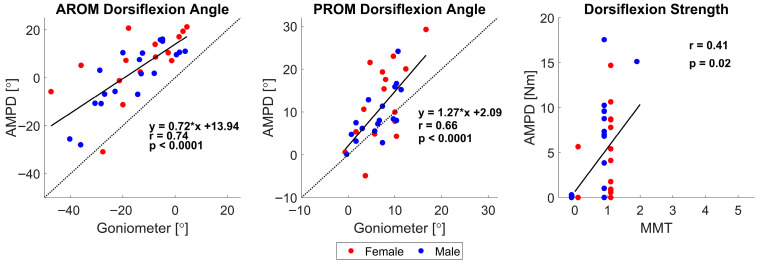
Relationships between clinical measures (*x*-axis) and AMPD measures (*y*-axis) of chronic stroke participants’ impaired ankles for active and passive ROM and maximum dorsiflexion strength. The dotted line represents a slope of 1, indicating perfect agreement between the two measures.

We analyzed how well dorsiflexion AROM, PROM, and MVC measurements taken with AMPD correlated with the same measurements taken by the therapist. We performed this analysis only for the post-stroke participants, as therapists did not obtain these measurements from the unimpaired participants. The correlations between robot- and therapist-obtained measures were strong for ROM and moderate for MVC (Figure 3).

However, the impaired ankle dorsiflexion ROM measured with the robot was larger than that with the goniometer (Figure 3, left and middle). Post-stroke participants were able to achieve, on average, 18.2 ± 9.9 degrees more dorsiflexion range of motion when the robot measured AROM (*p* < 0.0001) and 3.9 ± 6.0 degrees more when the robot measured dorsiflexion PROM (*p* < 0.0001), with a large effect size for both (d > 3.0).

As for maximum dorsiflexion strength, although the robotic and therapist measures of MVC were significantly correlated, the MMT scores had poor resolution. In total, 85% of participants scored 1 on the MMT, corresponding to “flicker or trace of contraction”. However, their ankle strengths were distributed across a range of 0 to nearly 20 Nm. Despite the coarse, ordinal nature of MMT, robotic MVC values were moderately correlated with MMT scores (r = 0.41, *p* = 0.02; Figure 3, right).

### 3.4. Reliability of Robotic Measures for Unimpaired Participants

We also used the robot to obtain the test–retest reliability of AROM and MVC for young and older unimpaired participants. Test–retest reliability was also good to excellent for these participants: ICCs were always 0.90 or above (Table 5).

### 3.5. Comparison of First- and Second-Session Measurements

As described above, the test–retest reliability of ankle impairment was high, though not perfect, raising the question of whether participants improved between the first and second testing sessions. For stroke participants, dorsiflexion measured with AMPD did not change significantly from the first to the second trial (*p* > 0.5, Table 3). For young and older participants, however, dorsiflexion AROM significantly increased for both young and older unimpaired participants by 1.3° ± 3.2° and 1.2° ± 2.4° (*p* < 0.001, Table 3), but it had a small effect (d < 0.20). Plantarflexion AROM and dorsiflexion MVC measurements did not change significantly between sessions for any group (*p* > 0.3, see Table 3).

### 3.6. Comparison of Older to Younger Unimpaired Adults and Effect of Leg Dominance

Robotic measurements revealed no significant differences in ankle dorsiflexion AROM (*p* > 0.1) or MVC (*p* > 0.5) between young and older participants. In young participants, the dominant ankle exhibited greater dorsiflexion AROM compared to the non-dominant ankle (*p* < 0.04), an effect not observed in the older group (*p* = 0.52). Both young and older participants demonstrated significantly greater dorsiflexion MVC in the dominant ankle compared to the non-dominant ankle (*p* < 0.001; see Figure 2 and Table 3), with medium to small effects (d < 0.25).

## 4. Discussion

We confirmed the hypothesis that a sensorized, robotic platform could provide reliable and valid measurements of ankle motor function after stroke. The reliability of both the ROM and MVC measurements was high for both the robot and the therapist, as well as for both the stroke and unimpaired groups. Further, the robot measurements for ROM were strongly correlated with therapist measurements and detected the significant effect of stroke and leg dominance on ankle function. The robot MVC measurements also showed higher granularity. However, the robot ROM measurements significantly differed from those of the therapists, an important consideration. We will now discuss these results, followed by limitations and directions for future research.

### 4.1. Considerations in Using Sensorized, Robotic Platforms as Ankle Assessment Tools

The motivation for this study was to evaluate the validity and reliability of a class of technology, sensorized robotic platforms, for the purpose of ankle function measurement. The robot we used, AMPD, was custom-built, but it is similar to several other research [37,38,39] and commercial [40] devices that can measure ankle function. The common features of these devices are that they test ankle function in a seated posture and use movement and force sensors integrated with a rotating foot plate strapped to the foot. This approach standardizes the testing posture, supports/constrains ankle movement (as opposed to the therapist having to manually do this), and provides fine-resolution measurements of angle and force. AMPD is mainly unique in its use of a mechanical method to switch between isometric force and free-motion measurements, but the overall measurement principles, standardized positioning, isolated joint testing, and integrated sensing are shared across this class of devices, supporting the broader relevance of our findings.

The high test–retest reliability of the robotic assessment of AROM and MVC across all groups, at a level comparable with that of the therapists, supports the concept that robotic devices could be useful for ankle function assessment. The reliability was not better than that of a therapist, but neither was it worse. Previous studies have found therapists to have moderate to good test–retest reliability for ankle range of motion [9,41,42]. Likewise, the finding of high test–retest reliability of MMT is consistent with those obtained in other studies for older [25,43] and young unimpaired adults [10].

The robotic assessments were not only reliable but also showed strong correlations with traditional therapist-based measurements, demonstrating concurrent validity. However, important differences emerged. Specifically, both active and passive range of motion (AROM and PROM) values were consistently higher when measured with the robotic device. This discrepancy could arise from several factors.

First, the robot may have held the foot in a more consistently pronated position than the therapist, which may allow the ankle to achieve greater angles of dorsiflexion ROM [44]. Second, the footplate used with AMPD contacted the plantar surface of the foot, which may have increased cutaneous input, a factor known to enhance force output [45]. Third, and most probable in our opinion, the discrepancy could have stemmed from the robot constraining the ankle to move strictly in the dorsiflexion/plantarflexion plane. In doing so, it blocks off-axis movements, allowing contributions from coupled or compensatory motions to be redirected into the primary movement plane. People with hemiparesis after stroke often lose the ability to move their joints independently, resulting in abnormally coupled pathophysiological movement patterns, also called synergies [16,46]. Ankle dorsiflexion is part of the so-called flexion synergy [47], and if the robot blocked the other components of this flexion synergy that occur at the hip, knee, and ankle, it may have allowed participants to leverage the synergy to dorsiflex the ankle further. This distinction should be kept in mind when interpreting robotic platform measurements and relating them to unconstrained, functional movements.

In the context of this study, the robotic approach provided greater granularity of strength measurement compared to the most common clinical method used here, the MMT. While continuous clinical measurement tools are available, the MMT remains the standard in most rehabilitation settings, and our comparison reflects this common practice rather than all possible clinical approaches. Individuals with the same MMT score created dorsiflexion torques that ranged from 0 to ~20 Nm. This highlights the robotic device’s ability to detect differences in strength that are obscured by the coarse, ordinal nature of the manual muscle test. Beyond increased granularity, continuous measurements such as those obtained from the robotic platform offer fundamental statistical advantages over categorical scales like the MMT. Continuous data better support parametric analyses, provide improved sensitivity to change, and enable richer modeling of motor recovery trajectories, making them more suitable for clinical research and outcome evaluation [48]. Future studies should perform comparisons with more accurate clinical methods, such as digital manual muscle testing. This would allow for the determination of how important the robot support for leg position is for repeatable measurements.

### 4.2. Bilateral Strength Deficits After Stroke

Here, we found that the dorsiflexion torque on the “unimpaired” side of stroke participants was significantly lower than that of age-matched controls. There has been a large body of work showing that unilateral stroke often produces bilateral motor consequences: subtle weakness and control deficits emerge in the contralesional (“unimpaired”) limb due to hemisphere-specific control changes and interhemispheric imbalance, even when clinical exams appear normal [49,50]. This has been demonstrated across tasks and cohorts, including lower-limb strength studies showing marked impairment in both limbs compared with controls [51,52]. Potential contributors include reduced descending neural drive and abnormal interhemispheric inhibition/corticomotor excitability [49,50,53,54], abnormal interlimb coupling/synergy that alters motor output bilaterally [52,55], learned non-use/decreased overall activity [56,57], and disuse atrophy and stroke-related sarcopenia [58,59,60], which compound age-related muscle loss. Together, these neural and musculoskeletal factors plausibly explain why the limb designated “unimpaired” nonetheless exhibits lower dorsiflexion torque than control limbs, highlighting the need to robotically evaluate both sides post-stroke.

### 4.3. Limitations and Directions for Future Research

This study has several limitations. First, we did not assess AROM in unimpaired participants using a goniometer, so test–retest reliability and validity analyses for AROM were limited to individuals post-stroke. Second, most stroke participants in our sample had severe ankle weakness, with MMT scores below 2; future studies should include individuals with milder impairments to assess generalizability. Third, our comparisons involved only two therapists. Although both therapists had over 20 years of experience and were highly trained, it remains possible that results could vary depending on the specific techniques used by the testing therapist [9,61]. Further, less skilled therapists may produce different results. Fourthly, a fixed ordering of therapist assessments followed by robotic assessments for stroke participants was used, and the test order of the individual components of the assessments was not randomized across participants either. This lack of randomization may have introduced potential fatigue or learning effects, which should be addressed in future studies. We note that both the therapist and robot assessments reported here involved only 1–3 trials, which reduces the likelihood that fatigue or learning played a role. Also, completing the therapist assessment before the robotic assessments had the advantage of preventing therapist scoring from being influenced by knowledge of robotic measurement results. Fifthly, therapists were not formally blinded to robotic measurements. AMPD’s interface displayed real-time performance feedback during robotic tests. To reduce potential bias, therapist assessments were always completed prior to the robotic assessments, which limits the likelihood that therapist scoring was influenced by robotic outputs, although full blinding was not implemented. Sixthly, inter-operator and inter-site reliability were not evaluated; thus, the reported ICCs reflect same-operator, same-device conditions and may differ under multi-rater clinical deployment.

Finally, it is important to note that using a robot for ankle assessment did not eliminate the need for human involvement. A trained experimenter or therapist was required to set up the control interface, position the participant, provide instructions and feedback, and monitor data quality. These human-controlled elements, including variations in participant positioning, strapping, and instruction delivery, could have contributed to the variability observed in the robotic measurements, and they should be considered in the interpretation of results. The robot serves not as a replacement for clinical expertise, but as an advanced measurement tool akin to a more sophisticated, sensorized goniometer that enhances, rather than replaces, human judgment.

## 5. Conclusions

We established that a sensorized, robotic platform can be highly reliable for evaluating ankle ROM and MVC, similar to a skilled therapist with more than 20 years of experience. In addition, robotic measurements of ROM demonstrated strong validity against therapist-based assessments, while MVC measures showed moderate validity despite the limitations of the ordinal MMT scale. Such an approach can also provide more granular strength assessments than the traditional therapist-based method examined here. A dataset for benchmarking future research is provided in Supplementary Materials. We hope that the supplementary dataset of ankle ROM and MVC values for post-stroke, age-matched, and younger individuals can serve as a useful benchmark for future research into ankle function.

## Figures and Tables

**Table 1:** Characteristics of stroke participants (N = 34).

	Average ± SD	[Min–Max]
Age	60 ± 12	[27 78]
Days Post-Stroke	1138 ± 1027	[201 4085]
[31] NIH Stroke Severity Scale [0 42]	6 ± 3	[2 16]
[32] Lower Extremity Fugl Meyer [0 34]	20 ± 4	[12 28]
[33] Modified Ashworth Score [0 4]	1.56 ± 0.46	[0 2]
[34] 6 min walk distance (meters)	107.9 ± 66.2	[0.20 298.50]
[35] 10 Meter Walk Test (m/s)	0.36 ± 0.24	[0 0.76]
[36] Montreal Cognitive Assessment [0 30]	22 ± 6	[1 30]
Ischemic/Hemorrhagic/Both	16/16/2	

**Table 2:** Characteristics of unimpaired participants.

	Number of Participants	Age [Min–Max]	Sex	Dominance
Young	36	25 ± 4 [19 33]	23M/13F	34R/2L
Older	26	64 ± 10 [50 84]	10M/16F	22R/4L

**Table 3:** Average dorsiflexion and plantarflexion active range of motion (AROM) and dorsiflexion maximum voluntary contraction (MVC) values from Sessions 1 and 2 for each ankle, stratified by group, side dominance, and sex. A: all participants, F: female, and M: male.

	Young		Old		Stroke	
	Dom.	Non-Dom.	Dom.	Non-Dom.	Impaired	Unimpaired
AROM Dorsiflexion Angle [°]						
Session 1	A: 25.04° ± 5.8	A: 23.8° ± 6.0°	A: 23.2° ± 6.0°	A: 23.0° ± 6.1	A: 2.7° ± 7.8°	A: 25.8° ± 8.2
	F: 24.8° ± 6.4°	F: 23.7° ± 5.2°	F: 22.8° ± 5.6°	F: 21.6° ± 6.3°	F: 5.6° ± 14.2°	F: 27.6° ± 8.6°
	M: 25.2° ± 5.6°	M: 23.8° ± 6.5°	M:24.0° ± 6.7°	M: 25.2° ± 5.4°	M: 0.4° ± 14.7°	M: 24.4° ± 7.9°
Session 2	A: 26.4° ± 5.9°	A: 25.0° ± 6.4°	A: 24.7° ± 7.0°	A: 24.0° ± 6.7°	A: 3.3° ± 13.6°	A: 25.5° ± 7.5°
	F: 25.8° ± 6.4°	F: 25.1°± 7.1°	F: 24.9° ± 7.8°	F: 23.1° ± 7.0°	F: 5.6° ± 14.6°	F: 25.7° ± 8.4°
	M: 26.7° ± 5.7°	M: 24.9° ± 6.2°	M: 24.3° ± 5.8°	M: 25.4° ± 6.3°	M: 1.5° ± 12.9°	M: 25.4° ± 6.9°
Change in mean	A: 1.3° ± 2.7°	A: 1.2 °± 3.7°	A: 1.3° ± 2.8°	A: 1.0° ± 2.1°	A: 0.6° ± 7.8°	A: −0.3° ± 2.7°
	F: 1.0° ± 3.5°	F: 1.3° ± 4.7°	F: 2.1° ± 2.9°	F: 2.1° ± 2.9°	F: 0.0° ± 7.6°	F: −1.9° ± 4.2°
	M: 1.5° ± 2.2°	M: 1.1° ± 3.1°	M: 0.4° ± 1.6°	M: 0.2° ± 2.5°	M: 1.1° ± 8.1°	M: 1.0° ± 4.1°
AROM Plantarflexion Angle [°]						
Session 1	A: −49.8° ± 6.3°	A: −48.5° ± 5.5°	A: −50.5° ± 6.3°	A: −48.7° ± 7.6°	A: −27.9° ± 10.5°	A: −47.2° ± 8.0°
	F: −50.2° ± 5.9°	F: −49.5° ± 5.3°	F: −51.9° ± 5.5°	F: −51.1° ± 6.9°	F: −28.7° ± 13.2°	F: −50.3° ± 9.4°
	M: −49.6° ± 6.6°	M: −48.0° ± 5.7°	M −48.2° ± 7.1°	M: −45.0° ± 7.4°	M: −27.2° ± 8.0°	M: −44.8° ± 5.8°
Session 2	A: −49.75° ± 7.0°	A: −48.3° ± 6.2°	A: −49.2° ± 5.3°	A: −48.6° ± 6.1°	A: −28.1° ± 12.0°	A: −46.8° ± 8.4°
	F: −49.8° ± 7.6°	F: −49.9° ± 6.1°	F: −50.3° ± 5.3°	F: 50.1° ± 6.1°	F: −27.0° ± 15.6°	F: −49.4° ± 10.3°
	M: −49.7° ± 6.8°	M: −47.4° ± 6.2°	M: −47.3° ± 5.0°	M: −46.2° ± 5.5°	M: −28.9° ± 8.5°	M: −44.7° ± 6.0°
Change in mean	A: 0.1° ± 2.8°	A: 0.2° ± 3.4°	A: 1.3° ± 3.3°	A: 0.1° ± 3.2°	A: −0.2° ± 6.4°	A: 0.4° ± 3.5°
	F: 0.4° ± 2.9°	F: −0.4° ± 3.0°	F: 1.6° ± 3.6°	F: 1.0° ± 3.1°	F: 1.7° ± 6.8°	F: 0.8° ± 3.3°
	M: −0.1° ± 2.8°	M: 0.5° ± 3.6°	M: 0.9° ± 2.9°	M: −1.2° ± 3.0°	M: −1.7° ± 5.7°	M: 0.0° ± 3.7°
Dorsiflexion Maximum Strength [Nm]						
Session 1	A: 25.9 ± 7.0	A: 27.0 ± 7.6	A: 20.9 ± 7.7	A: 22.9 ± 8.7	A: 4.9 ± 5.1	A: 20.1 ± 7.1
	F: 21.1 ± 5.2	F: 21.8 ± 5.5	F: 16.9 ± 3.6	F: 17.6 ± 3.9	F: 4.7 ± 4.6	F: 15.5 ± 4.9
	M: 28.6 ± 6.4	M: 30.0 ± 7.1	M: 27.3 ± 8.3	M: 31.4 ± 7.4	M: 5.1 ± 5.6	M: 23.8 ± 4.6
Session 2	A: 25.9 ± 7.1	A: 27.1 ± 7.1	A: 21.3 ± 6.9	A: 23.2 ± 8.9	A: 4.3 ± 4.9	A: 19.1 ± 6.9
	F: 21.7 ± 6.2	F: 22.0 ± 6.1	F: 18.5 ± 4.2	F: 18.9 ± 4.8	F: 4.7 ± 5.4	F: 15.3 ± 4.9
	M: 28.2 ± 6.6	M: 29.9 ± 6.0	M: 25.9 ± 8.0	M: 30.0 ± 9.8	M: 4.0 ± 4.6	M: 22.1 ± 6.9
Change in mean	A: 0.02 ± 2.7	A: 0.05 ± 3.0	A: 0.4 ± 3.5	A: 0.2 ± 3.9	A: −0.6 ± 3.2	A: −1.0 ± 3.9
	F: 0.6 ± 2.4	F: 0.2 ± 2.1	F: 1.6 ± 2.8	F: 1.3 ± 2.6	F: 0.1 ± 2.7	F: −0.2 ± 2.7
	M: −0.3 ± 2.9	M: −0.02 ± 3.5	M: −1.4 ± 3.9	M: −1.4 ± 5.1	M: −1.1 ± 3.5	M: −1.6 ± 4.6

**Table 4:** For both AMPD (R) and physical therapist (T), the ICC intraclass correlation coefficient, SEM standard error of measurement, and MDC minimal detectable change for the average of 3 trials for dorsiflexion AROM. For dorsiflexion MVC, only the first trial was used. This table only includes post-stroke participants.

	Stroke		
Trial	ICC [95% CI]	SEM	MDC
AROM Dorsiflexion Angle (Impaired Ankle)			
Avg. of 3	R: 0.92 [0.84 0.96]	R: 2.2°	R: 6.2°
	T: 0.95 [0.90 0.98]	T: 1.3°	T: 3.7°
Dorsiflexion MVC (Impaired Ankle)			
First	R: 0.89 [0.78 0.94]	R: 1.0 Nm	R: 2.9 Nm
	T: 0.86 [0.72 0.93]	T: 0.1	T: 0.3 levels

**Table 5:** Summary of test–retest reliability of AMPD. ICC intraclass correlation coefficient, SEM standard error of measurement, and MDC minimal detectable change for an average of 3 trials.

Young			Older			Stroke		
ICC [95% CI]	SEM	MDC	ICC [95% CI]	SEM	MDC	ICC [95% CI]	SEM	MDC
AROM Dorsiflexion Angle								
0.91 [0.85 0.95]	0.9	2.6	0.96 [0.90 0.98]	0.5	1.4	0.96 [0.94 0.98]	1.2	3.5
AROM Plantarflexion Angle								
0.94 [0.90 0.96]	0.6	1.8	0.93 [0.87 0.96]	0.9	2.5	0.96 [0.94 0.98]	1.0	2.7
Dorsiflexion MVC								
0.96 [0.94 0.98]	0.6	1.6	0.95 [0.91 0.97]	0.9	2.4	0.96 [0.94 0.98]	0.7	1.8

## Data Availability

The data presented in this study is available upon request from the corresponding author; however, robotic data is provided in the Supplementary Materials.

## Supplementary Materials

The following supporting information can be downloaded at https://www.mdpi.com/article/10.3390/s25206405/s1: Table S1: A supplementary dataset of robotic ankle ROM and dorsiflexion torque for unimpaired and chronic stroke participants, for benchmarking future research.
